# Combination therapy with cannabidiol and chemotherapeutics in canine urothelial carcinoma cells

**DOI:** 10.1371/journal.pone.0255591

**Published:** 2021-08-05

**Authors:** Jordon M. Inkol, Samuel E. Hocker, Anthony J. Mutsaers

**Affiliations:** 1 Department of Clinical Studies, Ontario Veterinary College, University of Guelph, Guelph, Ontario, Canada; 2 Department of Clinical Sciences, College of Veterinary Medicine, Kansas State University, Manhattan, Kansas, United States of America; 3 Department of Biomedical Sciences, Ontario Veterinary College, University of Guelph, Guelph, Ontario, Canada; Columbia University, UNITED STATES

## Abstract

**Background:**

Canine urothelial carcinoma is the most common form of canine bladder cancer. Treatment with chemotherapy has variable response rates leading to most dogs succumbing to their disease within a year. Cannabidiol is an emerging treatment within the field of oncology. In reported *in vivo* studies, cannabidiol has induced apoptosis, reduced cell migration, and acted as a chemotherapy sensitizer in various human tumor types. The aim of this study was to characterize the effects of cannabidiol on canine urothelial carcinoma cell viability and apoptosis as both a single agent and in combination with chemotherapy *in vitro*.

**Results:**

Cannabidiol reduced cell viability and induced apoptosis in canine urothelial cells as determined by crystal violet viability assay and annexin V/propidium iodide flow cytometry. Furthermore, combinations of cannabidiol with mitoxantrone and vinblastine chemotherapy yielded significantly reduced cell viability and increased apoptosis compared to single agent treatment alone. The drug interactions were deemed synergistic based on combination index calculations. Conversely, the combination of cannabidiol and carboplatin did not result in decreased cell viability and increased apoptosis compared to single agent treatment. Combination index calculations suggested an antagonistic interaction between these drugs. Finally, the combination of the non-steroidal anti-inflammatory drug piroxicam with cannabidiol did not significantly affect cell viability, although, some cell lines demonstrated decreased cell viability when mitoxantrone was combined with piroxicam.

**Conclusions:**

Cannabidiol showed promising results as a single agent or in combination with mitoxantrone and vinblastine for treatment of canine urothelial carcinoma cells. Further studies are justified to investigate whether these results are translatable *in vivo*.

## Introduction

Canine urothelial carcinoma (UC), also known as transitional cell carcinoma, is the most common form of bladder neoplasia in dogs. Common clinical signs include hematuria, dysuria, and stranguria [1]. Canine UC poses several clinical challenges due to its highly invasive nature, common anatomical location within the trigone region of the urinary bladder, and clinical presentations that are very similar to urinary tract infections/cystitis [1,2]. The most common treatment modalities for canine UC include chemotherapy, radiation therapy, non-steroidal anti-inflammatory drugs (NSAID), or combinations of these treatments. Complete surgical resection of the primary mass is often not feasible due to the trigonal location of the mass near urethral and ureteral openings [3]. Given the frequent lack of surgical treatment options and increased accuracy of tumor targeting with computerized radiation planning and conformal delivery, radiation therapy is being increasingly applied to treat canine urothelial carcinoma [4]. However, a large proportion of cases are managed with systemic therapy to treat the primary tumor mass. Objective response rates for conventional cytotoxic chemotherapy treatment of canine UC are variable, ranging from 0–58% [4–6]. Further, the overall median survival time for dogs that respond to chemotherapy is relatively short-lived at 242 days from the start of drug treatment [6,7]. Outside of conventional chemotherapy, the single agent use of NSAIDs remains a palliative treatment option for canine UC that may induce stable disease in many cases, with 3–20% of dogs experiencing a complete or partial tumor response [1,8]. When combined with chemotherapy, NSAIDs improve progression free interval and partial or complete response rates resulting in a marked clinical benefit compared to chemotherapy alone [9–11]. However, despite these improvements in outcome, it is still a low percentage of dogs that experience complete responses to treatment.

An emerging cancer therapy within the field of oncology is combination therapies of conventional treatments with cannabidiol (CBD). CBD is a phytocannabinoid derived from the *Cannabis sativa* plant with well-documented analgesic, anti-inflammatory, and anxiolytic effects [12–14]. Unlike other cannabinoids, CBD does not typically bind to cannabinoid receptors 1 (CB1) and 2 (CB2) [15–19]. Rather, CBD targets receptors such as transient receptor potential cation channel subfamily V receptor 1 (TRPV1), G-protein coupled receptor 55 (GPR55), and peroxisome proliferator-activated receptor gamma (PPARγ). In human oncology studies, CBD has been shown to induce tumor cell apoptosis through these non-canonical receptors, among others [15–19]. Furthermore, CBD has been shown to induce apoptosis through an autophagy-apoptosis crosstalk pathway orchestrated by Beclin1. Additionally, in human breast cancer, CBD attenuated cancer cell migration in human glioma [20,21]. This ability to induce cell death through two distinct mechanisms while having a large therapeutic index has generated interest in combining CBD with chemotherapeutic agents to improve their efficacy. Potential drug combinations for human breast and prostate cancer have been explored with results demonstrating that CBD enhanced the anti-tumor effects of chemotherapy at lower doses both *in vitro and in vivo* [22,23].

It is currently unknown if the reported beneficial anti-neoplastic effects of CBD are applicable to canine urothelial carcinoma. Our current study aimed to evaluate the single agent effects of CBD on canine urothelial carcinoma cell lines *in vitr*o and determine whether CBD can enhance the efficacy of common chemotherapy and NSAID drugs used to treat this disease.

## Materials and methods

### Cell lines and cell culture conditions

Three canine UC cell lines, AXA, Orig, and SH were utilized for this study and were kindly provided by Dr. Deborah W. Knapp at Purdue University. AXA and Orig were isolated from primary tumors and SH was isolated from a metastatic lymph node. Prior xenograft analysis confirmed AXA, Orig, and SH to be of canine UC origin [8]. All cell lines were cultured in Dulbecco’s Modified Eagle Medium (DMEM) with 10% Fetal Bovine Serum (FBS), 100 U/mL of penicillin/streptomycin (Thermo Fisher Scientific), and 5ug/mL of plasmocin (InvivoGen). Cells were tested mycoplasma negative before experimentation and maintained in a humidified incubator at 5% CO_2_ and 37°C.

### Chemical reagents

CBD was obtained from MediPharm Labs and resuspended at a stock concentration of 50 mM in a solution containing 5% ethanol, 5% Tween, and 90% Phosphate-Buffered Saline. Vinblastine and Carboplatin (Accord Healthcare) were obtained from the Ontario Veterinary College pharmacy and maintained at their stock concentrations of 2 and 10 mg/mL, respectively, in isotonic solution. Piroxicam and Mitoxantrone were purchased from Sigma-Aldrich and resuspended at a stock concentration of 50 mM and 20mM with dimethyl sulfoxide, (DMSO; Sigma-Aldrich) respectively.

### Crystal violet viability assay

The crystal violet assay was used to determine the half-maximal inhibitory concentration (IC50) for all drugs tested. Following initial cell growth kinetic/doubling time studies for each cell line, AXA, Orig, and SH were seeded at 2 x 10^4^, 1.5 x 10^4^, and 1.5 x 10^4^ cells per well, respectively, in 96-well plates and incubated for 16 hours. Once cells were adherent, medium was replaced with medium containing 0.1% FBS. Each cell line was treated with increasing doses of CBD (0.03–300 μM), vinblastine (0.01–10 μM), mitoxantrone (0.003–30 μM), carboplatin (0.01–1 mM), or piroxicam (0.01-1mM) for 24 hours. The cells were fixed with 0.5% crystal violet in 20% methanol (Thermo Fisher Scientific), and after drying overnight the dye was eluted with 10% acetic acid. Absorbance was read at 590 nm in triplicate and normalized to each drug’s respective vehicle control. IC50 curves were fit by a variable slope Hill’s equation and averaged by experiments conducted in triplicate.

### Combination Index (CI)

Drug combinations were performed for CBD with vinblastine, mitoxantrone, or carboplatin and compared to single agent treatment. Cell lines were treated with five drug concentrations alone or in combination for 24 hours. Doses were chosen at 0.125x, 0.25x, 0.5x,1x, and 2x times the value of their respective IC50. Cells were fixed with 0.5% crystal violet in 20% methanol (Thermo Fisher Scientific). Once dry, the dye was eluted with 10% acetic acid and absorbance was read at 590 nm. Each drug pairing’s CI value were calculated using CompuSyn (CompuSyn Inc). The Chou-Talay algorithm was used to simulate drug combinations and generate dose-reduction indices.

### Enhancement analysis

When a drug does not elicit a dose response curve, it cannot be used in combination analysis using the Chou-Talalay method. However, its potential effect on another drug can still be measured using enhancement analysis. Due to its lack of impact on cell viability as a single agent, drug enhancement analysis was elected for combinations of the NSAID piroxicam with vinblastine, mitoxantrone, carboplatin, or CBD. Cells were seeded at 2 x 10^4^, 1.5 x 10^4^, and 1.5 x 10^4^ cells per well for AXA, Orig, and SH, respectively, in 96-well plates and incubated for 16 hours. Once cells were adherent, media was replaced with media containing 0.1% FBS for dose response studies. Each cell line was treated with increasing doses of vinblastine (0.01–10 μM), mitoxantrone (0.003–30 μM), carboplatin (0.01–1 mM), CBD (0.03–300 μM) or with respective vehicle control for 24 hours. Based upon pharmacokinetic data, piroxicam was used at a dose of 20 μM [24]. Cells were stained with 0.5% crystal violet in 20% methanol (Thermo Fisher Scientific) and eluted with 10% acetic acid; absorbance was read at 590 nm. IC50 curves were compared via two-way ANOVA and Log IC50’s were compared via Student’s t test. All experiments were conducted in triplicate.

### Annexin V/Propidium iodide flow cytometry

Apoptotic response to treatment was quantified using fluorescein isothiocyanate (FITC)-conjugated annexin V and propidium iodide (PI; eBioscience). All cell lines were treated with either single agent or combination drugs for 24 hours in 0.1% FBS media prior to staining with annexin V-FITC (1:400) for 5 minutes and PI for 10 minutes at room temperature (RT). Cells were treated with 100 nM doxorubicin for 24 hours as a positive control for apoptosis. Collected conditioned media and cell fractions were run on a BD Acuri C6 flow cytometer (BD Biosciences) with a threshold of 5 x 10^4^ events per group. Laser intensities were based upon control readings and each data set was compensated to account for spillover between FITC and PI (FL1 and FL3 respectively). Early apoptosis was defined as annexin V positive and PI negative, while late apoptosis was defined as annexin V positive and PI positive.

### Statistical and software analysis

CompuSyn (CompuSyn Inc) was used for computation and simulation of combination indices. GraphPad Prism 8 (GraphPad Software Inc) was used for statistical testing and normality was assumed for linear or nonlinear regressions, unpaired t tests, and one-way or two-way ANOVA. Statistical significance was set at a *P* value of less than 0.05 and all means were reported with SD of the mean. FlowJo V10 (FlowJo LLC) was used for flow cytometry analyses.

## Results

### Effect of CBD and chemotherapy on canine UC cell viability

**Fig 1:**
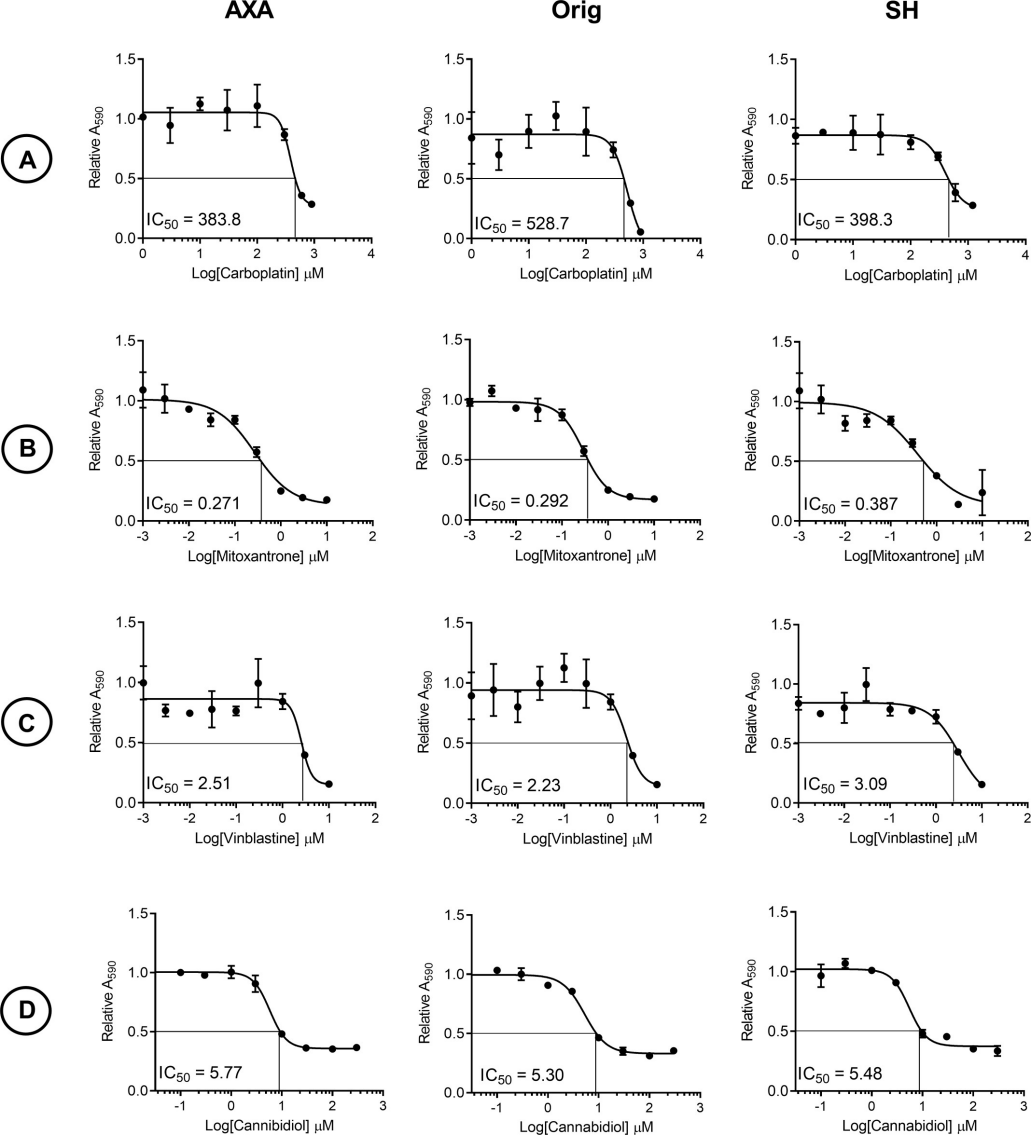
Dose-dependent decreases in cell viability with carboplatin, mitoxantrone, vinblastine, and CBD treatment of canine UC cells. Representative IC50 curves of cells treated with increasing doses of (A) carboplatin, (B) mitoxantrone, (C) vinblastine, and (D) CBD for 24 hours. IC50 values from graphs represent mean ± SD of three technical replicates and three biological replicates from each cell line.

Carboplatin (383, 528, and 420 μM), vinblastine (2.54, 2.19, and 2.25 μM), and CBD (5.77, 5.30, and 5.48μM) treatment yielded IC50 values in the micromolar range for AXA, Orig, and SH (Fig 1A, 1C and 1D). In contrast, mitoxantrone treatment yielded IC50 values in the nanomolar range (271, 282, and 362 nM) for AXA, Orig, and SH respectively (Fig 1B).

### Combination of CBD with chemotherapeutics

**Fig 2:**
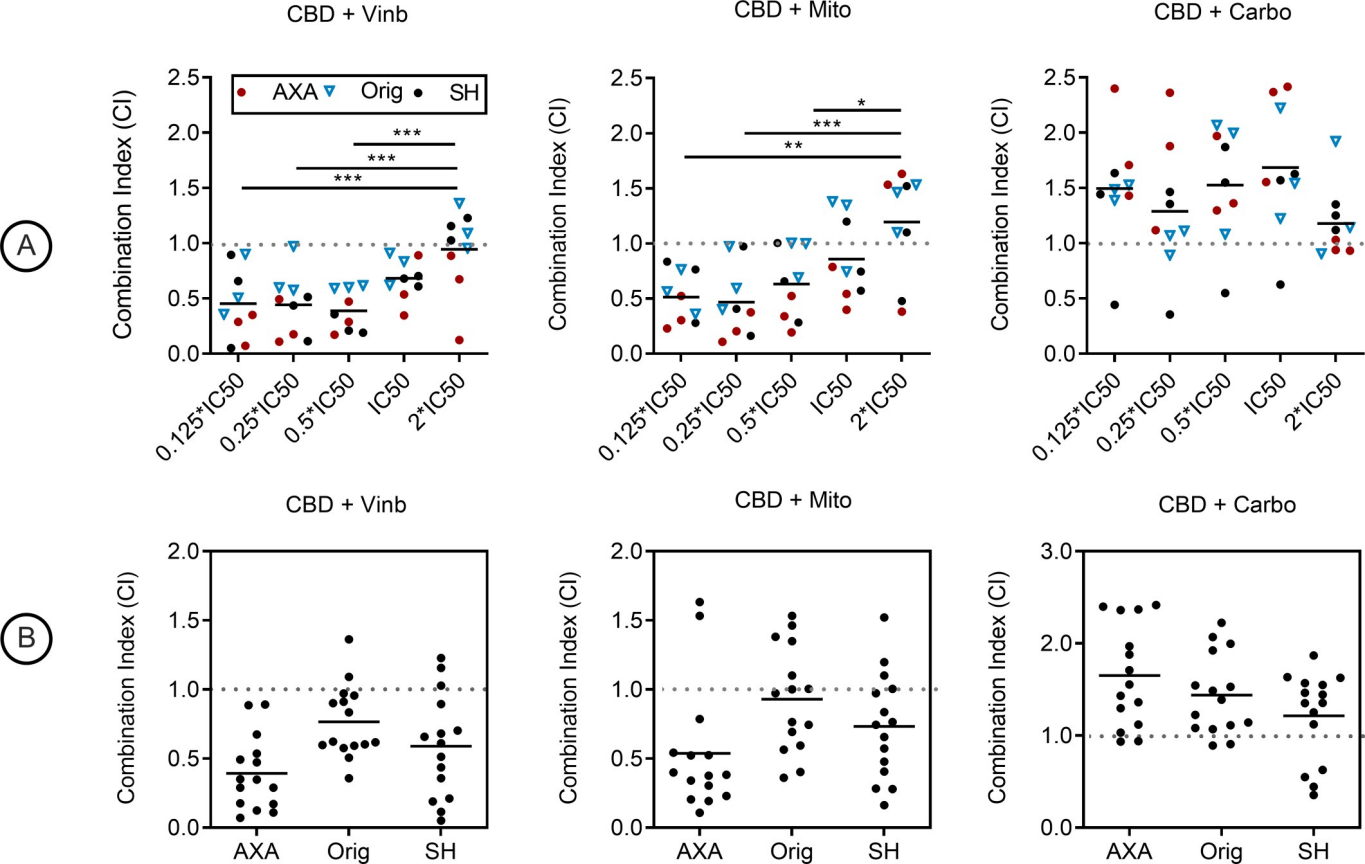
Combination treatment of CBD with mitoxantrone, vinblastine, or carboplatin in canine urothelial carcinoma cell lines. Combination index (CI) calculations in AXA, Orig, SH treated with CBD and vinblastine, CBD and mitoxantrone, or CBD and carboplatin. Dose pairings were chosen from respective IC50 curves. (A) Mean CI values from 3 independent experiments; stratified by dose-pairing. CI < 1 was synergistic, C = 1 was additive, and C > 1 was antagonistic. Significance was recorded at *P < 0.05, **P < 0.01, ***P < 0.001. (B) Mean CI values stratified by cell line independent of dose pairings.

Pharmacological interaction between CBD and vinblastine, as well as CBD plus mitoxantrone was observed to be synergistic (CI < 1). In the CBD plus vinblastine combination, the lowest mean CI observed was 0.39 ± 0.19 at the 0.5 IC50 dose pairing, which was significantly lower than the 2X IC50 dose pairing with a mean CI of 0.94 ± 0.37 (*P* < 0.001, Fig 2A). Significance was also recorded at *P* < 0.001 for 0.125 and 0.25 IC50 dose pairings (Fig 2A). The lowest observed CI for CBD plus mitoxantrone was 0.47 ± 0.32 for the 0.25 IC50 dose pairing (Fig 2A). The 0.125, 0.25, and 0.5 IC50 dose pairings were significantly lower than the 2X IC50 dose pairing which had a CI of 1.19 ± 0.47 (*P* < 0.01, *P* < 0.001, and *P* < 0.05, Fig 2A). However, CBD plus carboplatin yielded pharmacological antagonism (CI > 1) at each tested dose pairing (Fig 2A). Mean CI values stratified by cell line regardless of dose pairing were 0.30 ± 0.19, 0.77 ± 0.29, and 0.57 ± 0.38 for CBD plus vinblastine and 0.54 ± 0.49, 0.92 ± 0.42, and 0.67 ± 0.40 for CBD plus mitoxantrone in AXA, Orig, and SH cells, respectively (Fig 2B). CBD plus carboplatin mean CI values were 1.66 ± 0.55, 1.44 ± 0.44, and 1.21 ± 0.49 (Fig 2B).

### Enhancement analysis of piroxicam in combination with chemotherapy

**Fig 3:**
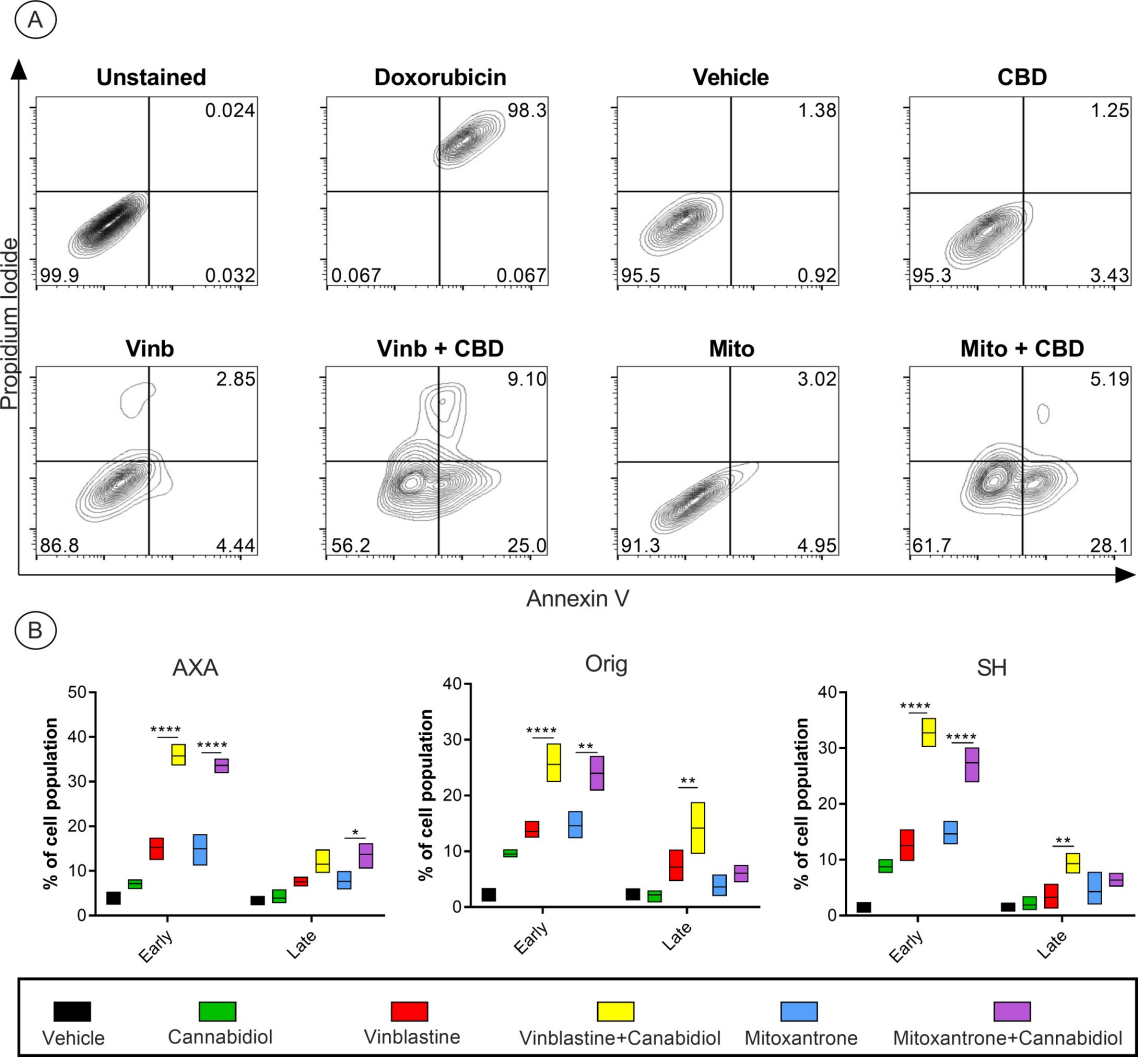
Piroxicam may enhance the cytotoxicity of mitoxantrone in canine UC cell lines. (A) Representative IC50 curves of canine UC cell lines treated with increasing doses of mitoxantrone, cannabidiol, vinblastine, and carboplatin in combination of 20 μM piroxicam or respective vehicle control for 24 hours. Graphs represent mean ± SD of three technical and biological replicates. (B) Log IC50 interpolated from IC50 curves in combination with and without piroxicam are presented with median ± range (N = 9). Significance was reported at * P<0.05, ** P <0.1, *** P < 0.001, **** P<0.0001. UC, urothelial carcinoma; IC50, half-maximal inhibitory concentration, P, piroxicam; Mito, mitoxantrone; Vinb, vinblastine; Carbo, carboplatin.

Due to the prevalence of piroxicam administration in canine UC clinical management, its potential pharmacological interactions with chemotherapeutics and CBD were explored *in vitr*o. Enhancement analysis was employed as piroxicam only generated dose-response viability curves at supra-physiological doses (S1 Fig). Combination of 20 μM piroxicam plus mitoxantrone recorded significant enhancement at 0.3 μM (*P* < 0.001), 0.03 μM (*P* < 0.05), and 1.0, 0.3, 0.1 μM (*P* < 0.01, *P* < 0.001, *P* < 0.01) doses of mitoxantrone in AXA, Orig, and SH cells, respectively (Fig 3A). Combination of piroxicam with cannabidiol resulted in significance at 300 and 10 μM (*P* < 0.0001, *P* < 0.05) in AXA, 300 and 0.1 μM (*P* < 0.001, *P* < 0.01) in Orig, and 300 μM (*P* < 0.01) in SH cells. Vinblastine combinations resulted in significance at 10, 1, and 0.3 μM (*P* < 0.05, *P <* 0.001, *P* < 0.05) in AXA and 1.0 and 0.1 μM (*P* < 0.01 and *P* < 0.05) in Orig. Combinations with carboplatin only recorded significance in the Orig cell line at 600 and 3 μM (*P* < 0.05, *P* < 0.05) doses (Fig 3A). Piroxicam only significantly lowered the log IC50 of mitoxantrone in AXA and SH cells, *P* < 0.05 and *P* < 0.01, respectively (Fig 3B).

### Effect of CBD on apoptotic response to chemotherapy

**Fig 4:**
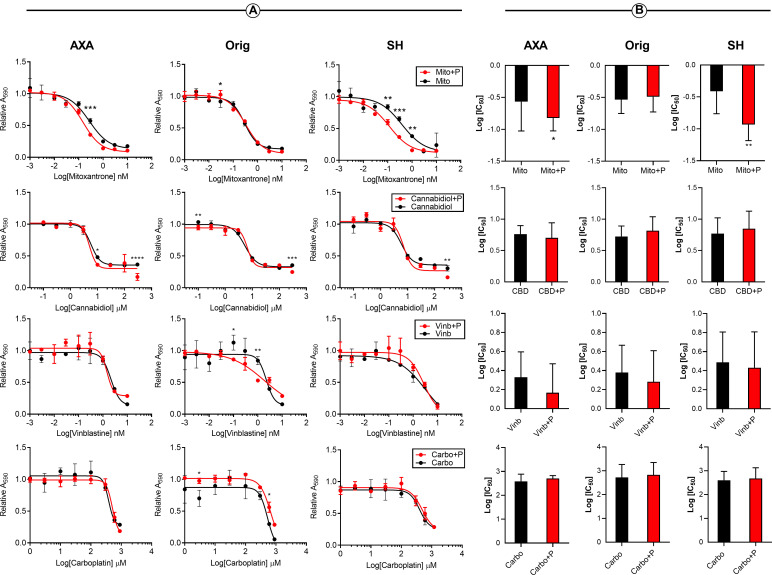
Combinations of CBD with mitoxantrone or vinblastine induces increased early apoptosis. Contour plots obtained after gating for singlets and spillover compensation (Fig 4A). Cell lines treated with a constant ratio of CBD, vinblastine, and mitoxantrone in single agent or combination treatment (0.5 IC50). Analysis of early apoptotic stages (Annexin V+/PI-) showed a significant increase in combination treatment compared to single agent in all cell lines (Fig 4B). Only Orig and SH showed a significant increase in late apoptosis for vinblastine + CBD. Mitoxantrone + CBD showed a significant increase in late apoptosis in AXA cells only (Fig 4A). Early and late apoptotic stages were presented as mean ± range from 3 independent experiments. Significance was reported at *P < 0.05, **P < 0.01, ***P < 0.001, ****P < 0.0001.

For early or late apoptosis only mitoxantrone and vinblastine were evaluated as carboplatin demonstrated antagonism when combined with cannabidiol in the viability studies. Single agent and combination treatment doses were selected at half of each respective drug’s IC50. Early and late-stage apoptosis was induced by both single agent and drug combinations (Fig 4A). Combination of vinblastine and cannabidiol induced a significant increase in early apoptosis in all cell lines compared to single agent treatments alone. When late apoptosis was examined, there was a significant increase in the combination treatment group compared to the single agent in both Orig and SH cell lines; however, this was not observed in AXA. CBD in combination with mitoxantrone treatment also yielded a significant increase in early apoptosis in all three cell lines. However, only the Orig cell line saw a marked increase in late apoptosis (Fig 4B).

### Potential drug interactions

**Fig 5:**
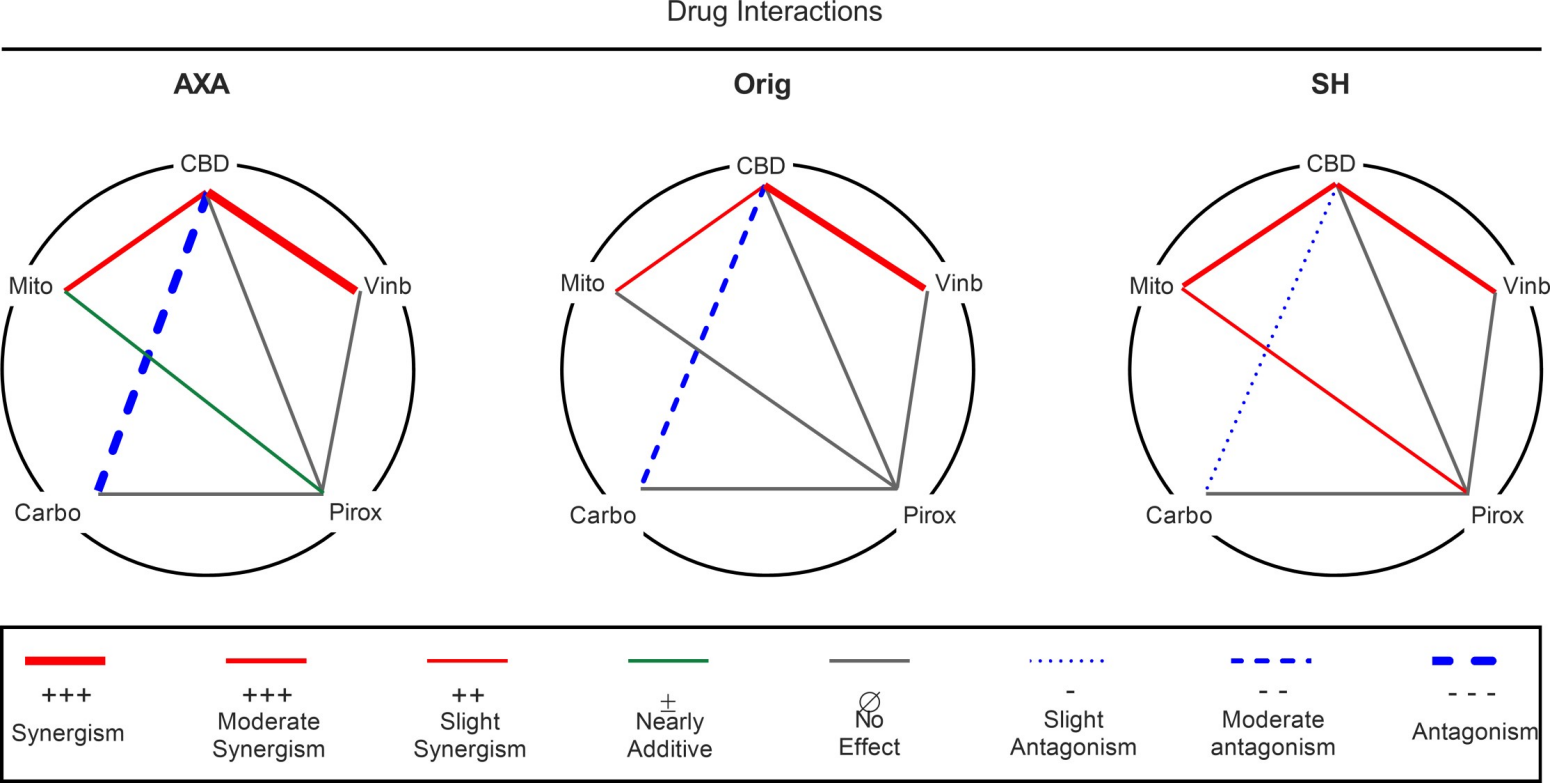
Polygonogram showing drug interactions of 5 drugs in combination with CBD or piroxicam in canine UC cell lines. Drug combinations are connected by lines, where synergism is indicated by solid red lines and thickness dictates the degree of synergism; heavy (CI = 0.1–0.5, synergism), medium (CI = 0.5–0.8, moderate synergism), and thin (CI = 0.8–0.9, slight synergism). Antagonism is indicated by blue broken lines, where thickness dictates degree of antagonism; heavy (CI >2.0, antagonism), medium (CI = 1.5–2.0, moderate antagonism), and thin (CI = 1.1–1.5, slight antagonism). Additive is indicated by a green line (CI = 1.0) and no effect is indicated by grey solid lines (no enhancement in cytotoxicity).

Interactions between cannabidiol, vinblastine, mitoxantrone, and carboplatin as well as piroxicam are summarized in a polygonogram (Fig 5). Pharmacological interactions were classified into eight categories based upon Chou’s methods [25]: synergism, moderate synergism, slight synergism, nearly additive, no effect, slight antagonism, moderate antagonism, and antagonism. Drug pairings are connected by colored lines: red indicates synergism, blue indicates antagonism, grey indicates no effect, and green indicates additive. Line thickness designates the strength of the relationship. Synergism (CI = 0.1–0.5) was only seen in AXA for CBD + vinblastine, while Orig and SH showed moderate (CI = 0.5–0.8) synergism for the same drug combination (Fig 5). Moderate synergism was shown in CBD + mitoxantrone for AXA and SH cells, while Orig demonstrated slight synergism (CI = 0.8–0.9). Carboplatin combinations produced antagonistic interactions in all cell lines. AXA displayed moderate antagonism (CI = 1.5–2.0), while both Orig and SH exhibited slight antagonism (CI = 1.1–1.5) (Fig 5). While almost all piroxicam combinations indicated no effect, piroxicam with mitoxantrone exhibited a nearly additive effect in AXA and SH cell lines.

## Discussion

As the potential anti-neoplastic effects of CBD are discovered and characterized in human oncology it is important to investigate whether these effects translate to veterinary oncology due to the need for novel and improved treatment modalities for pets with cancer. To the best of our knowledge, this study represents the first evaluation of pharmacological interactions between CBD and vinblastine, mitoxantrone, carboplatin, and piroxicam in the field of veterinary oncology. We demonstrated that CBD reduced urothelial carcinoma cell viability in a dose-dependent manner under low serum conditions, with an IC50 value that is achievable *in vivo* [26], suggesting that single agent CBD treatment alone has the potential to provide some therapeutic benefit for this disease. Based on Chou-Talalay derived combination indices, CBD demonstrated a synergistic interaction with both vinblastine and mitoxantrone, but an antagonistic interaction with carboplatin in canine UC cells (Fig 2). The favorable results with CBD and mitoxantrone are consistent with prior published studies involving similar drugs from the anthracycline class [27]. Furthermore, a mechanism that may potentially explain the synergistic combinations of both vinblastine and mitoxantrone is the ability of CBD to inhibit P-glycoprotein (P-gp), a drug efflux transporter responsible for multi-drug resistance that is induced by both anthracycline and vinca alkaloid chemotherapeutic families. Inhibition of P-gp results in greater accumulation of chemotherapy within the cell with subsequent enhancement of cytotoxicity [28,29]. However, it is also possible that other P-gp-independent mechanisms could also contribute to an observed synergistic effect, particularly as CBD acts as an agonist and antagonist of a multitude of cellular receptors.

After cell viability data and combination experiments suggested a synergistic interaction between CBD, mitoxantrone, and vinblastine, we sought to determine the effects of these drug combinations on early and late apoptosis (Fig 4). Both drug combinations resulted in marked increases in early apoptosis after 24 hours of incubation compared to vehicle and single agent treatment. Two potential mechanisms that may contribute to CBD-induced increases in apoptosis are autophagy and reactive oxygen species (ROS) generation. In a model of human breast cancer, CBD orchestrated crosstalk between autophagy and apoptosis. CBD increased LC3 expression while simultaneously increasing expression of truncated BH3 interacting-domain death agonist and procaspases [20]. This apoptotic cascade is unlikely to be initiated by the tumor suppressor gene and cell cycle control regulator p53 in Orig and SH, as both cell lines have minimal to no p53 expression [30]. However, while autophagy may lead to increased apoptosis, it may also conversely serve as a chemoresistance mechanism of cell preservation, as demonstrated in certain human tumor types [31]. Regarding ROS generation, CBD can induce substantial endoplasmic reticulum (ER) stress through ROS. This ER stress triggers intrinsic apoptosis, with ROS-Noxa being the dominant pathway [32].

In general, NSAIDs have been a consistent treatment option for dogs with UC to provide palliation and have recently been shown to confer a survival benefit when combined with chemotherapy [29]. One of the best studied NSAIDs for treatment of canine UC is piroxicam. We sought to explore the potential interaction between piroxicam and chemotherapy or CBD using enhancement analysis, as Chou-Talalay pharmacological interaction could not be performed due to a limitation in the method for drugs that do not show a consistent dose-dependent effect at biologically relevant concentrations. In order to determine synergism or antagonism each respective drug must be able to generate a unique dose-effect curve and potency [33]. Piroxicam generated a dose-effect curve at supra-physiological doses (IC50 > 1mM) (S1 Fig) which is consistent with other studies evaluating piroxicam *in vitro* [34]. Based on enhancement analysis, piroxicam potentiated the cytotoxic effects of mitoxantrone in AXA and SH 1.45 and 2.26-fold respectively; but not Orig (Fig 3B). A possible underlying mechanism for this response is unknown currently, as AXA is positive for COX2 while SH is negative for COX2 via western blotting. Furthermore, both cell lines have unknown COX1 expression, notwithstanding the fact that piroxicam may exert COX-independent effects [30,35]. Despite no other tested drug combinations demonstrating enhancement *in vitro*, when moved into clinical evaluation, NSAID-chemotherapy combinations can yield longer survival compared to their respective single agent treatment [9,36,37]. It is suggested that piroxicam does not necessarily contribute to direct cancer cell cytotoxicity but rather produces beneficial effects involving the tumor microenvironment (such as angiogenesis inhibition) that *in vitro* models cannot easily recapitulate [38].

There are some inherent limitations to this study. The first being the nature of the study being conducted *in vitro* which is unable to recapitulate cellular interactions and may not accurately reflect the drug interactions *in vivo* as a result. Further, serum conditions utilized in cell culture studies, including the low levels utilized in our experiments, may not accurately reflect the nutritional components of the tumor microenvironment.

## Conclusion

This study determined that CBD treatment reduced viability and induced cell death in canine urothelial carcinoma cells *in vitro*. In addition, when combined with mitoxantrone and vinblastine, CBD potentiated the apoptotic response generated by these chemotherapeutic agents in a synergistic manner. Combinations of piroxicam with vinblastine, mitoxantrone, carboplatin, and CBD showed that piroxicam potentially enhances the cytotoxicity of mitoxantrone *in vitro*. Taken together, these results suggest that CBD may be a potential treatment for use in combination with chemotherapeutic agents to improve canine UC carcinoma response rates and survival. Further studies *in vivo* are warranted, and clinical trials are necessary to investigate how best to implement CBD-chemotherapy combination treatments in a clinical setting.

## Supporting information

S1 Fig Piroxicam and vehicle control do not impact canine UC cells *in vitro*. Representative IC50 curves for piroxicam treatment of AXA, Orig, and SH cells show no dose dependent decrease in cell viability up to 1mM (A). Combination of Mitoxantrone and vehicle used for CBD (ethanol, TWEEN-20, and phosphate buffered saline) showed no significant effect on overall IC50 curve (B) or log IC50 value compared to mitoxantrone alone (C) in AXA cells. IND, indeterminate.
(TIF)

S2 Fig Low serum conditions do not alter background apoptosis levels. Contour plots of AXA cells under 10% and 0.1% fetal bovine serum conditions reveals no significant difference in background apoptosis levels.
(TIF)
