# Supplementary figures and images for: Combination therapy with cannabidiol and chemotherapeutics in canine urothelial carcinoma cells
Source: PLoS One. 2021 Aug 5;16(8):e0255591. doi: 10.1371/journal.pone.0255591 (PMC8341525; doi:10.1371/journal.pone.0255591)

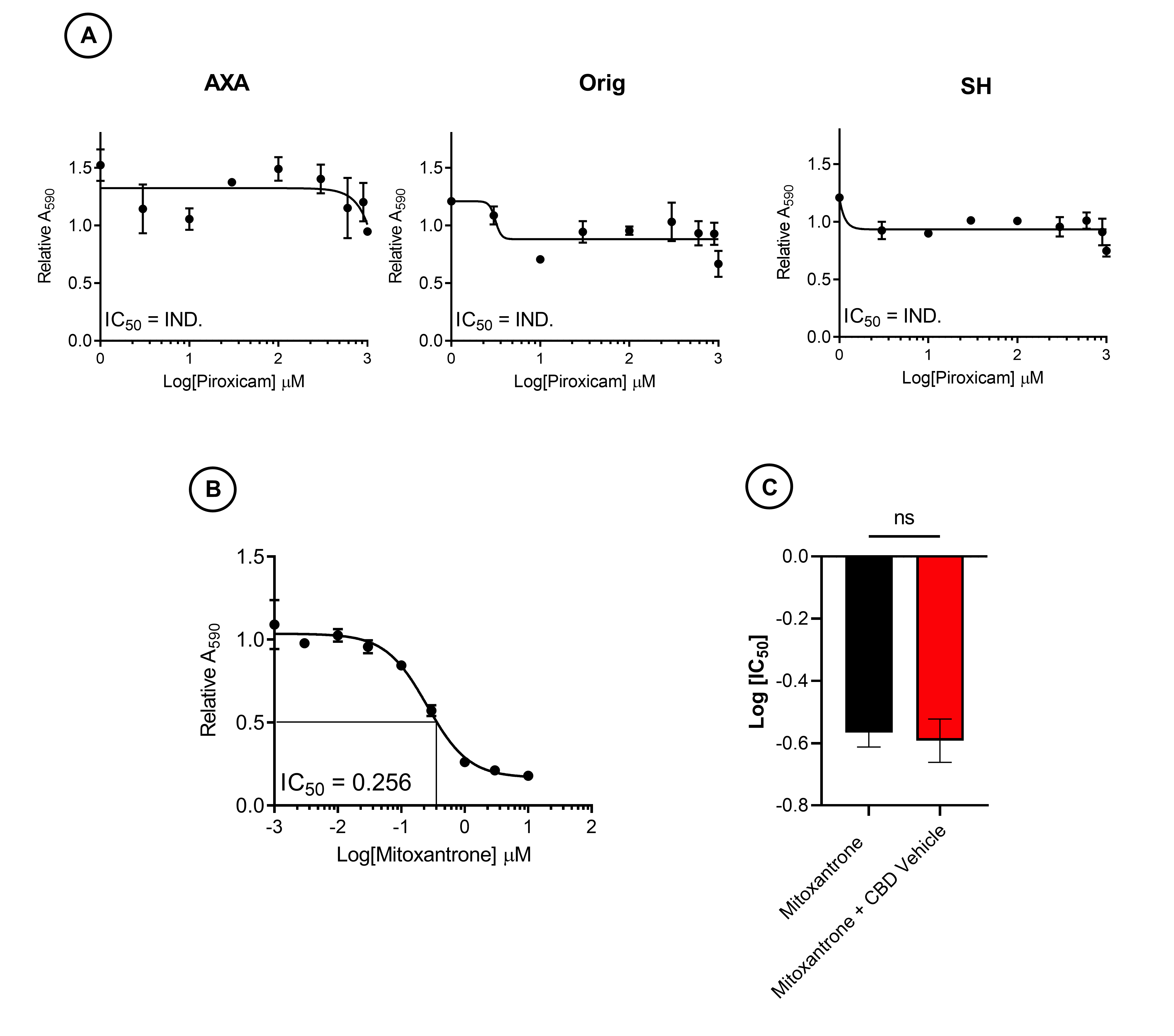

Supplement: S1 Fig — Representative IC50 curves for piroxicam treatment of AXA, Orig, and SH cells show no dose dependent decrease in cell viability up to 1mM (A). Combination of Mitoxantrone and vehicle used for CBD (ethanol, TWEEN-20, and phosphate buffered saline) showed no significant effect on overall IC50 curve (B) or log IC50 value compared to mitoxantrone alone (C) in AXA cells. IND, indeterminate. (TIF) [file pone.0255591.s001.tif]

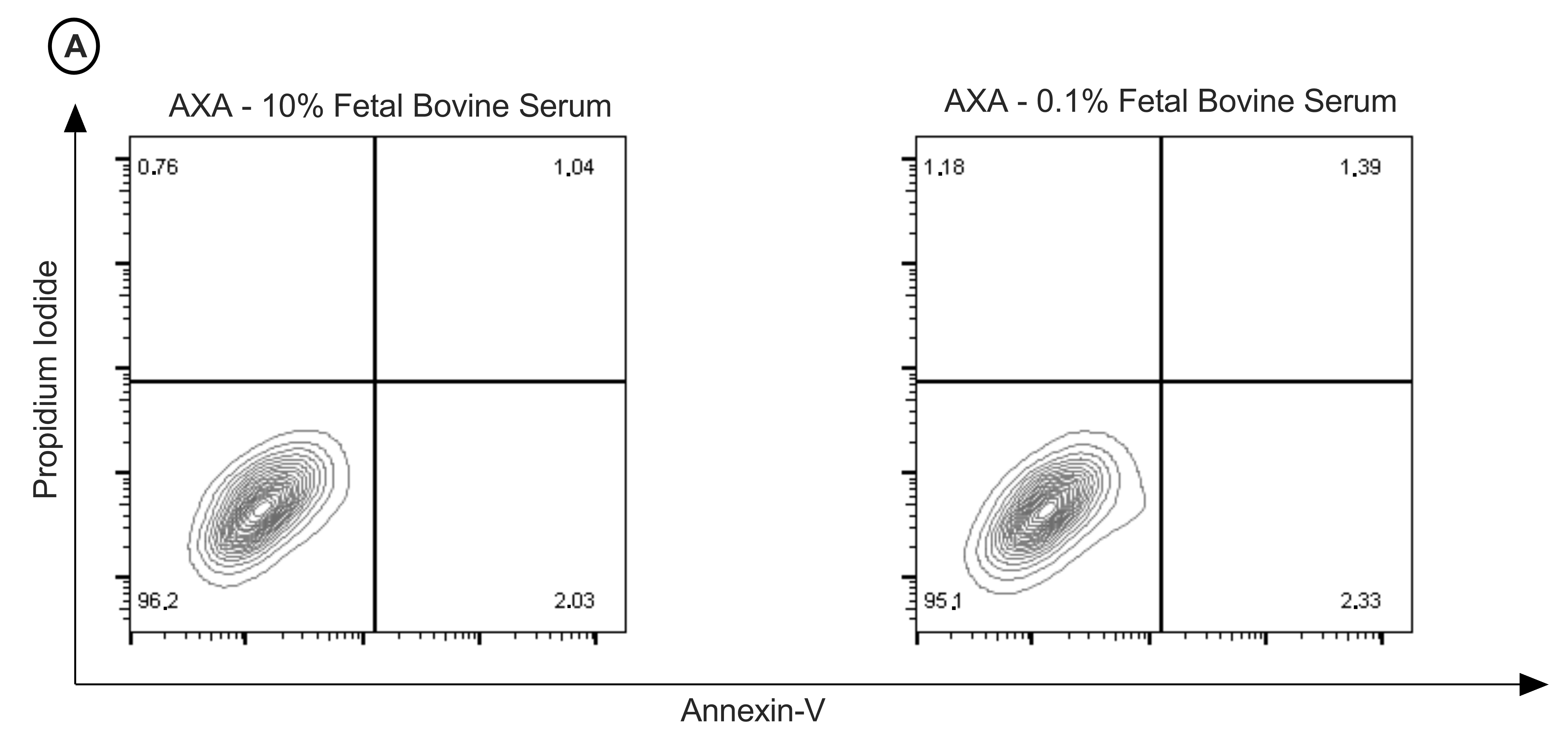

Supplement: S2 Fig — Contour plots of AXA cells under 10% and 0.1% fetal bovine serum conditions reveals no significant difference in background apoptosis levels. (TIF) [file pone.0255591.s002.tif]
